# KBE009: A Bestatin-Like Inhibitor of the *Trypanosoma cruzi* Acidic M17 Aminopeptidase with In Vitro Anti-Trypanosomal Activity

**DOI:** 10.3390/life11101037

**Published:** 2021-10-01

**Authors:** Jorge González-Bacerio, Irina Arocha, Mirtha Elisa Aguado, Yanira Méndez, Sabrina Marsiccobetre, Maikel Izquierdo, Daniel G. Rivera, Katherine Figarella, Néstor L. Uzcátegui

**Affiliations:** 1 Department of Biochemistry, Faculty of Biology, University of Havana, Havana 10400, Cuba; jogoba@fbio.uh.cu; 2 Center for Protein Studies, Faculty of Biology, University of Havana, Havana 10400, Cuba; mirtha.aguado@fbio.uh.cu (M.E.A.); mizquierdo@fbio.uh.cu (M.I.); 3 Laboratory for Genomics and Proteomics, Biotechnology Center, IDEA Foundation, Caracas 1080, Venezuela; iarocha@csleon.com (I.A.); Marsiccobetre@usp.br (S.M.); kfigarella@gmail.com (K.F.); 4 Center for Natural Products Study, Faculty of Chemistry, University of Havana, Havana 10400, Cuba; Yanira.MendezGomez@ipb-halle.de (Y.M.); dgr@fq.uh.cu (D.G.R.); 5 LaBTryps, Department of Parasitology, Institute of Biomedical Sciences, University of Sao Paulo, Sao Paulo 05508-000, Brazil; 6 Department of Neurophysiology, Institute of Physiology, University of Tuebingen, 72076 Tuebingen, Germany; 7 Immunochemistry and Ultrastructural Laboratory, Anatomical Institute, Universidad Central de Venezuela, Caracas 1053, Venezuela; 8 Institute of Tropical Medicine, University of Tuebingen, 72074 Tuebingen, Germany

**Keywords:** bestatin-like peptidomimetics, chemotherapy, leucyl-aminopeptidase, protease inhibitors, *Trypanosoma cruzi*

## Abstract

Chagas disease, caused by the kinetoplastid parasite *Trypanosoma cruzi*, is a human tropical illness mainly present in Latin America. The therapies available against this disease are far from ideal. Proteases from pathogenic protozoan have been considered as good drug target candidates. *T. cruzi* acidic M17 leucyl-aminopeptidase (TcLAP) mediates the major parasite’s leucyl-aminopeptidase activity and is expressed in all parasite stages. Here, we report the inhibition of TcLAP (IC_50_ = 66.0 ± 13.5 µM) by the bestatin-like peptidomimetic KBE009. This molecule also inhibited the proliferation of *T. cruzi* epimastigotes in vitro (EC_50_ = 28.1 ± 1.9 µM) and showed selectivity for the parasite over human dermal fibroblasts (selectivity index: 4.9). Further insight into the specific effect of KBE009 on *T. cruzi* was provided by docking simulation using the crystal structure of TcLAP and a modeled human orthologous, hLAP3. The TcLAP-KBE009 complex is more stable than its hLAP3 counterpart. KBE009 adopted a better geometrical shape to fit into the active site of TcLAP than that of hLAP3. The drug-likeness and lead-likeness in silico parameters of KBE009 are satisfactory. Altogether, our results provide an initial insight into KBE009 as a promising starting point compound for the rational design of drugs through further optimization.

## 1. Introduction

Chagas disease is a tropical illness present mainly in Latin America. However, in the last two decades, mostly due to human migrations, a significant increase in Chagas disease has been detected in North America, Europe, and the Western Pacific [1,2]. Its etiological agent is the kinetoplastid protozoan *Trypanosoma cruzi* (*T. cruzi*), responsible for ~50,000 new cases and ~10,000 deaths every year. About 6–7 million people are currently infected with the parasite [1,3]. Importantly, between 65–100 million people are living in regions at risk for infection around the world [2].

The sickness comprises two stages, which can progress differently in time [4,5]. The acute phase lasts four to eight weeks and is characterized by the presence of the parasite in the blood. In this phase, patients are usually asymptomatic or present non-specific signs and symptoms of infection such as fever, malaise, anorexia, lymphadenopathy, among others. Later, Chagas disease can become silent (the so-called indeterminate phase) which involves a long latency (habitually more than ten years) [4,5]. Finally, 20–30% of the infected people progress to the chronic phase, in which Chagas cardiomyopathy is the main complication but the brain or gastrointestinal organs are also commonly compromised [6,7].

The two drugs available for treatment, namely nifurtimox and benznidazole, are partially effective and significantly toxic [8]. They only work in the acute phase of the disease, which usually, unfortunately, goes unnoticed due to the absence of specific symptoms. Additionally, their efficacy is compromised by poor adherence to medication by patients as a consequence of the onset of adverse drug reactions. Effective treatment protocols to alleviate these adverse effects are missing [1]. Therefore, new and effective chemotherapies against Chagas disease are urgently required.

A rational drug design is based on the identification and characterization of new molecular targets. This approach can make a significant contribution to the fight against persistent sicknesses, which are often difficult to treat, as Chagas disease [8]. In this regard, proteases are considered good drug targets [9], as these enzymes are involved in many aspects of the parasite’s physiology [10,11].

**Figure 1:**
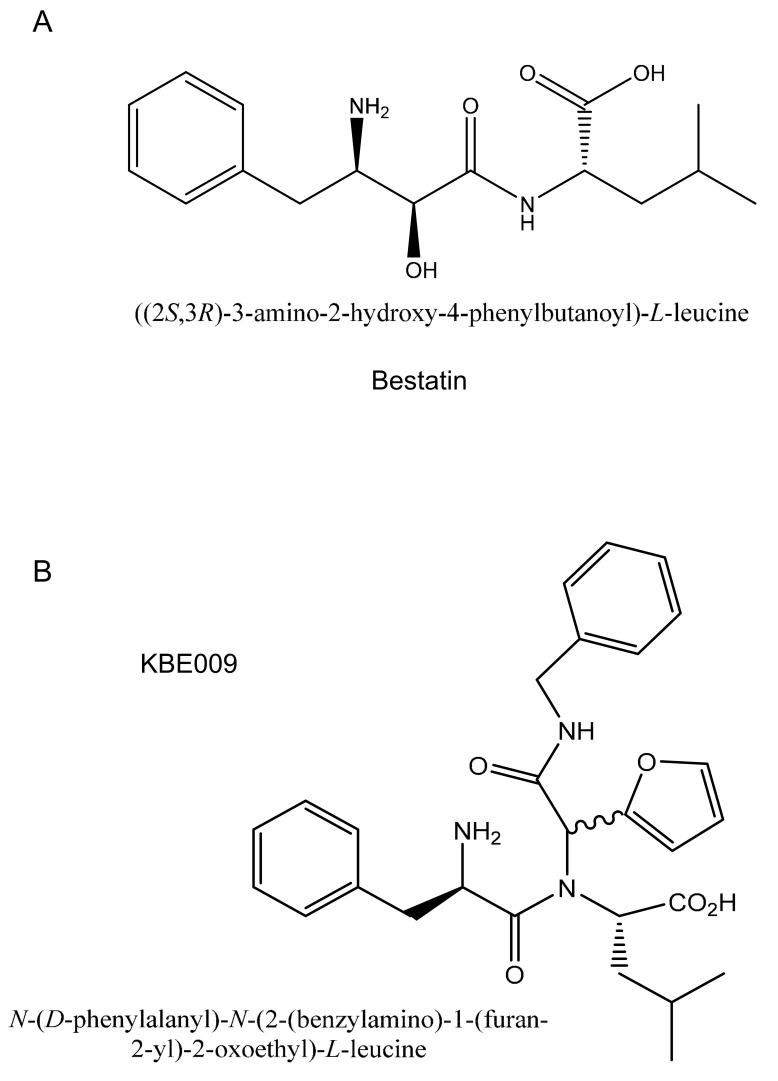
Molecular structure of the metallo-aminopeptidase inhibitors: (**A**) Bestatin; (**B**) KBE009. The s-shaped bond shows the stereocenter responsible for the generation of diastereomers.

Interestingly, *T. cruzi* possesses an acidic (according to isoelectric point) metallo-aminopeptidase belonging to the M17 family of proteases (TcLAP, also known as LAPTc in the literature). This enzyme mediates the main leucyl-aminopeptidase (LAP) activity in the parasite and is expressed in all parasite stages [12]. TcLAP may participate in nutritional supply since the parasite lacks the biosynthetic pathway for leucine. Interestingly, bestatin (Figure 1A), a classic inhibitor of metallo-aminopeptidases [13], causes in situ inhibition of TcLAP in *T. cruzi* epimastigotes [14]. Thus, inhibition of TcLAP by bestatin-like molecules may be a feasible strategy to develop anti-chagasic drugs.

Previously, it was reported that the bestatin-like peptidomimetic KBE009 (Figure 1B) is an inhibitor of *Plasmodium falciparum* M1 aminopeptidase (PfA-M1) and exerts antimalarial activity in vitro against 3D7 and FcB1 strains [15]. This compound is selective for the plasmodial enzyme over its porcine counterpart, and it is not cytotoxic against human umbilical vein endothelial cells (HUVECs).

Here, we initiated the study of KBE009 as a viable starting point for the rational development of molecules against *T. cruzi*. Our findings demonstrated that KBE009 can inhibit the native M17-type LAP activity in parasite protein extracts as well as the rTcLAP. KBE009 had a deleterious effect on the proliferation of *T. cruzi* epimastigotes, which was more prominent than that observed in human fibroblasts. In the searching for anti-Trypanosomal agents, these characteristics point to KBE009 as a promising compound. The structure of this molecule can be specifically optimized to comply with the hit-and-lead criteria for Chagas disease [16], and therefore pave the way for making progress towards a new effective drug against this disease.

## 2. Materials and Methods

In the literature, the acidic enzyme leucyl-aminopeptidase from *T. cruzi* has been abbreviated as LAPTc or TcLAP (both of them correct) [12,14,17,18]. However, here we denoted the enzyme as TcLAP, following the recommendations of the genetic nomenclature for *Trypanosoma* and *Leishmania* proposed at the Woods Hole Molecular Parasitology Meeting (Falmouth, MA, USA, 1996) as well as at the WHO-sponsored workshop for the *T. brucei* and *Leishmania* genome project (Arcachon, France, 1998) [19].

### 2.1. Comparison of the Amino Acid Sequence of TcLAP with Other Orthologous LAPs and Analysis of Their Phylogenetic Relationship

First, the TcLAP protein sequence (GN = Tc00.1047053508799.240) was used as a query for BLAST search on the NCBI website to find homologs sequences. Matched sequences with a query coverage higher than 80% and an e-value < e^−60^ were chosen for the phylogenetic tree to ensure the evolutionary relationship with TcLAP.

Subsequently, a phylogenetic tree of LAP sequences was constructed using the MEGA X Software (Molecular Evolutionary Genetics Analysis) (https://www.Megasoftware.net/, accessed on 17 March 2021) [20]. A sequence alignment was performed using MUSCLE (http://www.drive5.com/muscle, accessed on 17 March 2021) [21]. The results were employed to construct the LAP phylogenetic relationship using the ‘neighbor-joining tree’ algorithm of the MEGA X Software. Clusters were constructed containing four related sequences each. In the clusters with only three members, an additional sequence was selected. In this case, those with a coverage of at least 65% of the query and an e-value < e^−30^ were selected. A bootstrap analysis was carried out with 10,000 replicas to ensure good support in the estimation of clades.

To obtain the percent of amino acid identity among LAPs, 12 sequences used to construct the phylogenetic tree were chosen and compared through multiple sequence alignment using MUSCLE. The identity percent matrix was created with the Clustal2.1 algorithm from the EMBL-EBI server (https://www.ebi.ac.uk/Tools/msa/, accessed on 20 March 2021). To construct the multiple sequence alignment picture, the software Jalview 2.11.1.3 was used (http://www.jalview.org/development/release-history/Jalview-21113, accessed on 20 March 2021) [22].

### 2.2. Building of hLAP3 3D-Model

The crystal structure of the human leucine aminopeptidase 3 (hLAP3; GenBank: CAG33409.1) has not been published so far. Therefore, its structure was modeled using the swiss-model website (https://swissmodel.expasy.org/, accessed on 5 April 2021). First, several templates were obtained by a search in the swiss-model database using the hLAP3 sequence. Then, templates that possessed high sequence similarity to hLAP3 were chosen for 3D modeling. Considering several parameters such as the Global Model Quality Estimate (GMQE), the Quaternary Structure Quality Estimation (QSQE), crystal structure resolution, etc., the best 3D model of hLAP3 generated was selected. The best model was obtained using the bovine leucine aminopeptidase as a template (1LCP; Protein Data Bank [PDB]), which shares an amino acid sequence identity of 92.56% with hLAP3. Manipulation of the protein structure (e.g., representation, calculations, comparison) was performed using Chimera Software (www.rbvi.ucsf.edu/chimera/ accessed on 2 April 2021) [23].

### 2.3. Molecular Docking

Molecular docking experiments were carried out using Chimera and AutoDock Vina [24]. The crystal structures of TcLAP, without (apoenzyme) and with citrate (holoenzyme) (5NTF and 5NTG) were obtained from the PDB databank (http://www.wwpdb.org/, accessed on 2 April 2021). The hLAP3 structure was obtained by modeling as mentioned above. Protein receptors were prepared for docking as usual. KBE009 diastereomers (R and S) were first drawn as a 2D structure using the ChemDraw^®^ software (PerkinElmer). Then, using the Avogadro Software (https://avogadro.cc/, accessed on 26 March 2021) [25], the 3D structure, optimized geometry, and energy minimization of KBE009 (R, S) were obtained. Both KBE009 diastereomers (R and S) were employed for docking experiments, however, for simplicity, only KBE009 R was depicted in the figures. The docking area was defined empirically using a grid encompassing the enzyme’s active site. The grid dimension and its orientation were the same for both the protozoan and the human enzymes. The result was chosen considering the Auto Vina score, the lowest energy (kcal/mol) of the complex enzyme-KBE009 represents the most stable complex. The interactions between molecules (e.g., number of H-bonds) were also considered. Based on these criteria, the best two or three binding poses were chosen. Finally, the 3D graphic representation, as well as calculation of H-bond formation, van der Waals contacts, among others, was performed with Chimera (https://www.cgl.ucsf.edu/chimera/, accessed on 12 April 2021) [23].

### 2.4. TcLAP Aminopeptidase Activity Inhibition Assays

The generation of recombinant TcLAP (rTcLAP) was previously reported [17]. Its aminopeptidase activity was determined by a continuous kinetic method using the chromogenic Leu-p-nitroanilide (Leu-pNA) molecule as substrate (Bachem, Bubendorf, Switzerland). Leu-pNA was used at 75 µM (added from a 7.5 mM stock dissolved in DMSO, analytical grade, Sigma-Aldrich, St. Louis, MO, USA) and the increase of the absorbance at 405 nm (OD_405nm_), due to p-nitroaniline (pNA) chromogen formation, was recorded every 15 s over 5 min using a spectrophotometer (FLUOstar OPTIMA, BMG Labtech GmbH, Offenburg, Germany). This substrate concentration represents ~1 Km (apparent) for rTcLAP [17]. The determinations were carried out in enzymatic activity buffer (50 mM Tris-HCl pH 9.0, 4 mM CoCl_2_) at 50 °C, which is the optimal temperature of the recombinant enzyme [17]. The enzyme concentration was 9.09 × 10^−7^ M (measured by the bicinchoninic acid method), it was chosen from the linear range plotting initial velocity (V_0_) vs. enzyme concentration. The final volume of the reaction was 200 µL, and the measurement was performed in flat bottom 96-well plates. To measure the reaction velocity, exclusively the linear range of the typical curves was used. It corresponds to substrate consumptions lower than 5% (V_0_ conditions). Slopes with coefficients of determination (R^2^) < 0.98 were not considered for linear fits. The assays were carried out in triplicate.

For the inhibition assays, rTcLAP in an enzymatic activity buffer was mixed with different concentrations of KBE009 (6.25–200 µM range) [26]. The mixture was preincubated for 30 min at 50 °C before adding the substrate. KBE009 was dissolved in DMSO, analytical grade, and the DMSO final concentration used was not higher than 2% (*v*/*v*). All other experimental conditions were maintained as described above. Control reactions were performed by pre-incubation of the enzyme withw/v the solvent. Residual activity was defined as the quotient between the reaction velocity in the presence of KBE009 and the control. The IC_50_ values were calculated by the nonlinear fit of the logistic function to the experimental data using the GraphPad Prism software (version 8.0.2). The logistic function is:

y = 1/(1 + [I]/IC_50_), (1)

where y is the residual aminopeptidase activity, and [I] is the inhibitor concentration in the assay [27].

### 2.5. Obtainment of Protein Extracts from Epimastigotes of Trypanosoma cruzi CL Brener

Four 25 mL-parasite cultures in 10% bovine fetal serum (BFS)-supplemented Liver infusion tryptose (LIT) medium, at 2 × 10^6^ cells/mL, were prepared. These cultures were incubated at 25 °C for 5 days, the time at which the cultures are in the growth exponential phase and have 3 × 10^7^ cells/mL. Afterward, cells were collected by centrifugation at 2000× *g* for 15 min at 4 °C. Pellets were washed three times with 10 mL of cold PBS 1X, by centrifugation for 10 min at 2000× *g* and 4 °C, and resuspended in 1.5 mL of cold PBS 0.1X containing 20 µM of the protease inhibitors TLCK and E-64. Cell lysis was performed by ultrasonic treatment in a 2 mL-centrifuge tube with two pulses of 15% ampleness for 15 s each one, with intervals of 30 s on ice. The efficiency of the rupture step was confirmed by checking the cellular integrity with the optical microscope. The suspension was centrifuged for 20 min at 10,000× *g* and 4 °C, and the supernatant was transferred to clean tubes on ice.

### 2.6. M17-Type Aminopeptidase Activity Inhibition Assays in Protein Extracts from Epimastigotes of Trypanosoma cruzi CL Brener

The aminopeptidase activity was determined by a continuous kinetic method, similar to that described above. The determinations were carried out with 250 µM Leu-pNA substrate, in 50 mM Tris-HCl, 400 µM MnCl_2_, at pH 7.0 and 37 °C. The amount of protein extract was chosen in the linear range of the plot V_0_ vs. protein. For the inhibition assays, KBE009 (100 µM) was pre-incubated with the protein extract for 45 min at 25 °C before adding the substrate. Here again the final concentration of the solvent DMSO in the reaction mixture did not exceed 2%. Assays were performed in triplicate.

### 2.7. In Vitro Measurement of the Anti-Trypanosomal Activity of KBE009 against Trypanosoma cruzi Epimastigotes

Epimastigotes of *T. cruzi* CL Brener strain were cultivated in LIT medium (4 g/L NaCl, 0.4 g/L KCl, 8 g/L Na_2_HPO_4_, 2 g/L glucose, 5 g/L triptose, 5 g/L liver infusion, 25 mg/L hemin, 10% BFS filtrated with 0.22 µm filter). Cultures were incubated at 25 °C in a refrigerated incubator (Innova 4230, New Brunswick Scientific, Edison, NJ, USA) for six days maximum. Parasite density was determined by direct counting in the NeuBauer chamber using an optic microscope (MicroscopioOptika SRL, Ponteranica, Italy).

For the in vitro anti-Trypanosomal activity assay, serial dilutions in DMSO (cell-culture grade) of the molecule KBE009 in the 0.625–20 mM range were prepared. Epimastigotes in exponential growth phase were diluted to 2 × 10^6^ cells/mL in LIT medium and 200 µL per well of this suspension were transferred into a sterile 96-well microplate. From each KBE009 dilution, 2 µL were added into the wells to assay concentrations in the 6.25–200 µM range. Plates were incubated at 25 °C for five days, a time by which cultures are in the exponential growth phase and thus proliferation relies only on duplication.

After that time, cells were collected by centrifugation at 3000× *g* for 10 min at 25 °C, the supernatant was discarded by inversion, and pellets were resuspended in 100 µL Thiazolyl Blue Tetrazolium Bromide reagent (MTT, 0.5 mg/mL in PBS) (Sigma-Aldrich, St. Louis, MO, USA). Plates were incubated for 4 h at 37 °C and 5% CO_2_ (Thermo Electron Corporation, Hepa Class 100, Marietta, OH, USA). Then, cells were collected by centrifugation at 3000× *g* for 10 min at 25 °C, and the tetrazolium crystals formed were dissolved with 100 µL DMSO (technical grade, Sigma-Aldrich, St. Louis, MO, USA). Finally, absorbance was measured at 570 nm in a microplate spectrophotometer (Synergy HT, Biotek Instruments, Winooski, VT, USA) using the Gen 5 software (v. 1.11) supplied by the manufacturer. As control condition (100% growth), cultures exposed to 2 µL DMSO were used. To confirm that the solvent does not affect the proliferation rate, cultures without solvent were also included. A blank for each KBE009 concentration consisting of culture medium plus the molecule without parasites was included. Each condition was assayed by triplicate. The experimental data were processed according to Bodley et al., 1995 [28], and the EC_50_ value was calculated by the nonlinear fit of the logistic function using the GrapPadPrism software (version 8.0.2). Experiments were performed at least three times independently.

### 2.8. In Vitro Measurement of the KBE009 Cytotoxicity against Primary Cultures of Human Dermal Fibroblasts

Human dermal fibroblasts were cultured in RPMI medium (RPMI plus 10, Sigma-Aldrich, St. Louis, MO, USA) supplemented with 10% BFS, 200 mM glutamine, and penicillin-streptomycin, and allowed to grow in an incubator at 37 °C and 5% CO_2_ (Thermo Electron Corporation, Hepa Class 100, Marietta, OH, USA). When cultures reached confluence, cells were dissociated with trypsin in PBS. Then, cells were immediately resuspended in a culture medium and collected by centrifugation at 3000× *g* for 10 min and 25 °C. Cells were resuspended in fresh culture medium, counted in NeuBauer chamber, and cell density adjusted to 1 × 10^5^ cells/mL. Fibroblasts were transferred into a sterile 96-well microplate at 5000 cells/well and allowed to adhere for 24 h at 37 °C and 5% CO_2_.

Working solutions of KBE009 ranging from 0.3125 mM to 20 mM were previously prepared in DMSO (cell-culture grade). Thus, KBE009 was added to assay concentrations in the 3.125–200 µM range. Plates were incubated for three days at 37 °C and 5% CO_2_ to guaranty evident exponential growth phase. After that time, the medium was discarded by inversion, 100 µL of the MTT reagent (0.2 mg/mL in PBS) added, and plates were further incubated for 4 h at 37 °C and 5% CO_2_. The following steps were the same as described for *T. cruzi* CL Brener. Here, the corresponding controls and blanks were also included. Experiments were performed at least three times independently.

## 3. Results

### 3.1. TcLAP Is Quite Divergent and Does Not Share a High Identity with the hLAP

**Figure 2:**
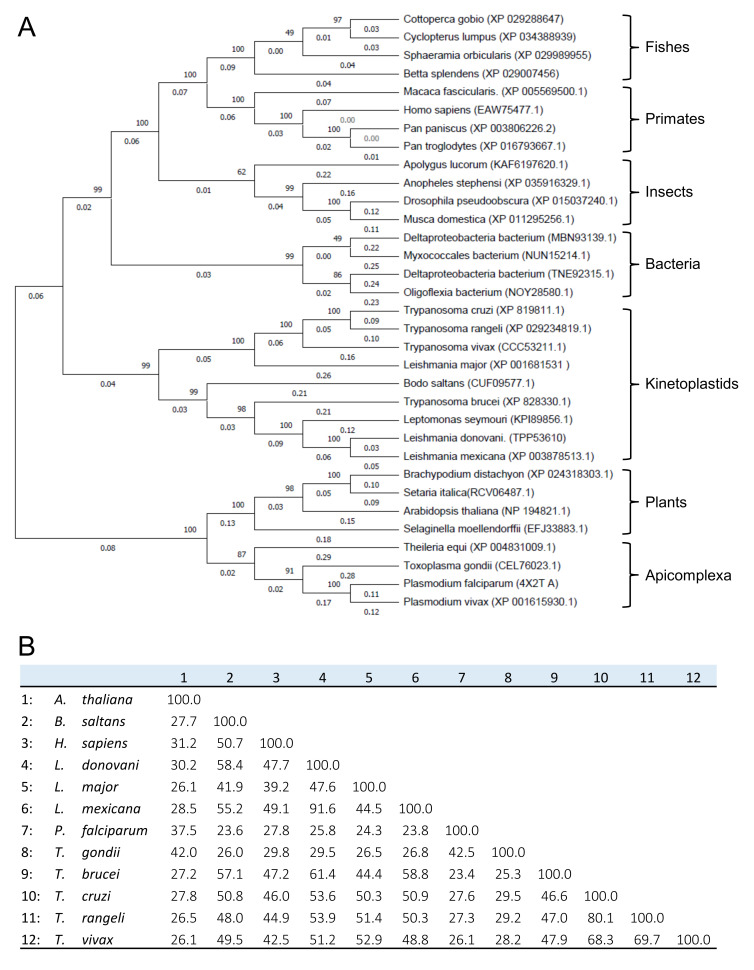
Phylogenetic analysis and amino acid sequence identity of LAPs: (**A**) phylogenetic tree of 33 full-length protein sequences chosen from the NCBI database. It was constructed with the neighbor-joining algorithm using MEGA X software. The identification of the sequences is in brackets. Numbers below and above of the line of the branches indicate the evolutionary distance between sequences and the bootstrap proportions in percentage (10000 replicates), respectively; (**B**) twelve LAP sequences used for the construction of the phylogenetic tree were compared. Multiple sequence alignment was performed using MUSCLE, and the percent amino acid identity was determined using Clustal2.1 on the EMBL-EBI server (https://www.ebi.ac.uk/Tools/msa/, accessed on 20 March 2021). The names of the species are the same as in the phylogenetic tree.

**Figure 3:**
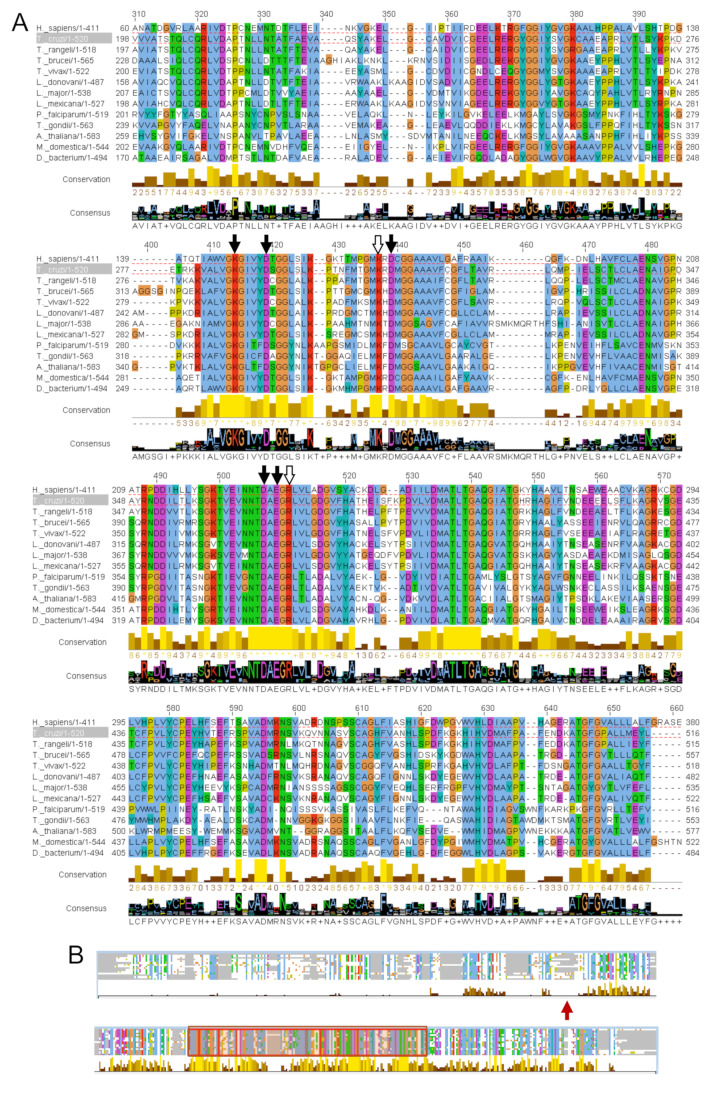
Amino acid sequence comparison of LAPs: (**A**) sequence alignment of the LAPs was produced using the MUSCLE approach by the Jalview software. Amino acids are highlighted according to the ClustaLx code. The non-conserved N-termini of sequences were not included in the image. Amino acid residues of the catalytic site and the putative metal-binding site are indicated above the alignment by white- and black-arrows, respectively; (**B**) schematic overview of the complete alignment. The red arrow indicates the starting region of the alignment represented in part A of the figure. The red rectangle shows the most conserved region.

A phylogenetic tree was constructed with 33 amino acid sequences of the LAPs using TcLAP for initial BLAST. With strong bootstrap support, the phylogenetic tree shows divergent groups of proteins distributed into eight clusters (Figure 2A). Kinetoplastids generated two highly close clades. As expected, *Bodo saltans* possesses the most distant sequence in these clades of Kinetoplastids as this non-pathogenic protozoon belongs to the suborder Bodonina, while the rest of the sequences come from the pathogenic protozoa belonging to the suborder Trypanosomatina [29]. Interestingly, LAPs of the Apicomplexa parasites are evolutionarily closer to LAPs from plants than those of trypanosomes. Notably, the TcLAP sequence is evolutionarily fairly divergent from the human one. Multiple amino acid sequence alignments of the LAPs showed that TcLAP shares a high identity with other trypanosome sequences. TcLAP is 80% and 68% identical to *T. rangeli* and *T*. *vivax*, respectively (Figure 2B). However, it shares moderate identity to the putative human LAP (46%) and less than 30% identity with other LAPs belonging to Apicomplexa parasites. As expected, a comparison of the LAPs confirmed that the C-terminal region is highly conserved while the N-terminal one is strongly dissimilar (Figure 3) [12].

The conserved amino acids that are part of domains for metal binding (K287, D292, D310, D369, and E371 in TcLAP sequence) and catalytic activity (residues K299 and R373 in TcLAP) are present in the C-terminus and are conserved across different taxa (Figure 3A).

### 3.2. The Bestatin Derivative Molecule KBE009 Inhibits the Activity of the Recombinant TcLAP and Native M17-Type LAP Activity in Parasite Protein Extracts

**Figure 4:**
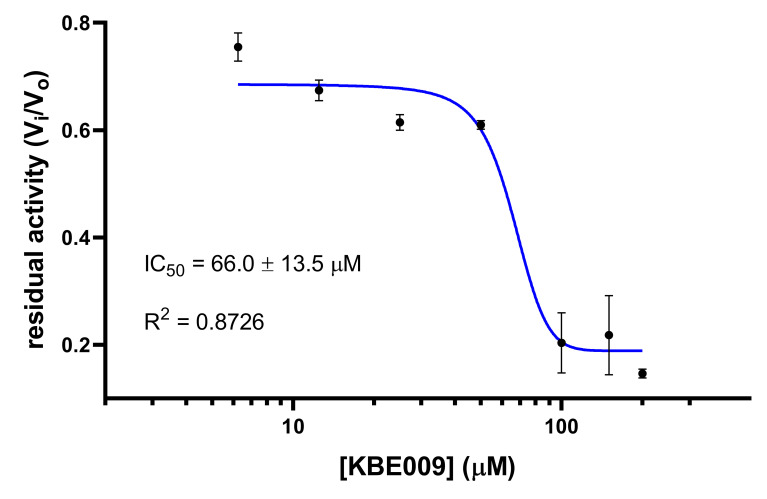
Dose-response inhibition of recombinant TcLAP by KBE009. KBE009 concentrations between 6.25 µM and 200 µM were used to evaluate the activity of the rTcLAP. The chromogenic substrate Leu-pNA was used at 75 µM. A sigmoidal fit was applied to the experimental values on the residual activity obtained by each concentration, and the IC_50_ was calculated using the GraphPad Prism software. Vi, velocity of the enzymatic reaction in the presence of the inhibitor; V_0_, velocity of the enzymatic reaction in the absence of the inhibitor. The squares on the plot represent the means with standard deviations for each KBE009 concentration determined at least three times.

The effect of KBE009 on the rTcLAP’s activity was evaluated immediately after its purification. Here, we used the protocols previously described by Izquierdo et al., 2019 [17]. To ensure proper inhibition, KBE009 was preincubated with the enzyme before the activity measurement started. Under the experimental conditions, we found that KBE009 inhibits the rTcLAP with an IC_50_ value of 66.0 ± 13.5 µM (Figure 4). Additionally, to confirm the inhibitory effect on the native enzyme, protein extracts from epimastigotes of *T. cruzi* (CL Brener strain) were prepared, and the M17-like aminopeptidase activity was detected using the chromogenic substrate Leu-pNA. Extracts were challenged with KBE009, obtaining an activity inhibition of 38.4 ± 5.8% when the peptidomimetic concentration was 100 µM.

### 3.3. KBE009 Inhibits the Proliferation of Epimastigotes of T. cruzi

**Figure 5:**
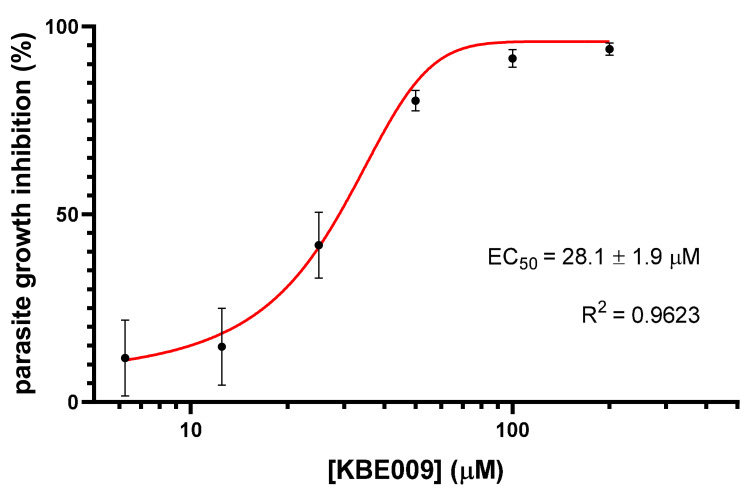
In vitro inhibition of the *T. cruzi* epimastigotes growth by KBE009. Epimastigotes were allowed to grow in the presence of different concentrations of the peptidomimetic KBE009. The effect on the culture growth was determined after five days of incubation. The graphic represents the results (mean ± standard deviation) of four independent experiments. The sigmoidal adjustment of the experimental values for determining the EC_50_ was fit using the GraphPad Prism software.

Due to the inhibiting effect of KBE009 on the TcLAP observed, we also investigated if the peptidomimetic could exert an action on the parasite growth in vitro. Epimastigotes were treated with different concentrations of the bestatin derivative for five days to determine its cytotoxic effect. As expected, a dose-dependent growth inhibition was obtained when concentrations between 6.25 µM and 200 µM were used. The concentration that inhibits half of the parasite’s proliferation (here referred as EC_50_ to differentiate it from the IC_50_ related to the inhibition of the enzyme activity) was determined to be 28.1 ± 1.9 μM (Figure 5). This concentration is about three times lower than the one required to inhibit the native M17-type LAP activity in parasite protein extracts.

Moreover, to test the cytotoxicity of KBE009 in mammalian cells, human dermal fibroblasts were also assessed. Here, again a dose-response inhibition of the fibroblast proliferation was observed. However, the EC_50_ value was 138 ± 10µM; it represents a concentration 4.9 times higher than the one needed to reach the same effect on the parasite growth. Due to experimental growth conditions and to guaranty reaching the exponential growth phase in each case, EC_50_ values were determined for epimastigotes and fibroblast after five and three days, respectively. Still, when parasites were incubated with 100 µM, they were unable to even duplicate after three days of incubation.

### 3.4. 3D Protein Structure of TcLAP and hLAP3 Reveals Differences at the N-Terminus

3D structure comparison and docking studies were performed to understand further the inhibitory effect of KBE009 on TcLAP and the possible differential action on the human aminopeptidase (hLAP3). The 3D structure of the hLAP3 was obtained using a bovine leucine aminopeptidase from *Bos taurus* as a template, which shares an amino acid sequence identity of 92.56% with the hLAP3 (for details, see materials and methods). A comparison of the overall 3D structure of the enzymes showed high similarities.

**Figure 6:**
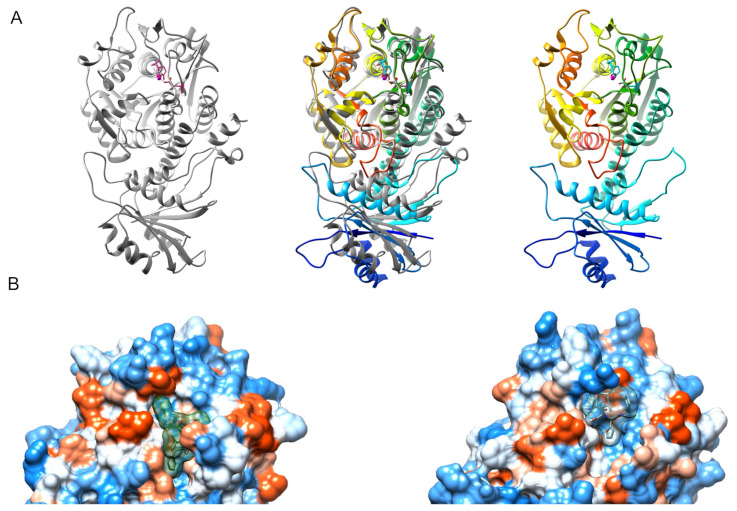
3D ribbon structures of TcLAP and hLAP3 and their predicted docking with KBE009: (**A**) tertiary structures of the monomer of TcLAP (left) and hLAP3 (right) alone, and both superposed (middle) to make evident distinctiveness. The rainbow colors in hLAP3 denote N- and C-termini as follows: blue/green (N-terminal), yellow/red (C-terminal); (**B**) docking estimation of TcLAP and KBE009 (left) and hLAP3 and KBE009 (right). Only the part of the monomer of the enzyme that possesses the catalytic pocket is shown. The surface of the enzymes represents the volume and the hydrophilicity/hydrophobicity of amino acid residues (blue hydrophilic and red hydrophobic). KBE009 appears in a stick, with a semi-transparent surface representing the volume of the compound. For simplicity, only one of the poses of R-KBE009 was represented. To ensure an equal protein orientation in this representation, TcLAP and hLAP3 were first overlaid, and then they were split into two panels for clarity. To facilitate the comparison, the side chains of some of the conserved amino acids in the metal-binding domains were represented (D292, D369, and E371 in TcLAP, and their counterpart in hLAP3; D364, D287, and E366).

The secondary structures, α-helices, and β-sheets are conserved through all proteins. The secondary structures of TcLAP and hLAP3 overlap at the C-terminal ends. However, it does not occur completely at the N-terminal regions of the enzymes (Figure 6A), and the differences increase as it is closer to the N-termini. This difference is due to the low conserved sequences at this region in these proteins, a general characteristic of the LAP enzymes [30].

### 3.5. KBE009 Docking Prediction Shows That the Peptidomimetic Molecule Bears More Affinity for TcLAP Than for hLAP3

TcLAP and hLAP3 were docked with both diastereomers of KBE009 (R, S) using the same protocol. The crystal structure from TcLAP and a modeled structure of the hLAP3 were used for docking. The best binding poses for each docking were chosen mainly according to scoring in autodock vina; the number of H-bonds and the similarities of geometrical fitting of the structures in the catalytic pocket were also considered. For the TcLAP/KBE009 (R), three poses with the highest binding energy (−7.5 kcal/mol) were selected. The superposition of all three KBE009 (R) conformations shows a similar pattern (Figure S1). The main difference lies in only one conformation, which overlaps with a 180-degree rotation, the other two conformations are almost equal. For TcLAP/KBE009 (S), two binding poses with the highest binding energy (binding energy −7.3 and −7.1 kcal/mol) were selected. The overlapping conformations are practically the same (Figure S1). When binding poses of R and S diastereomers are compared, the same pattern appears; all five conformations superimpose at the same place at the catalytic pocket and maintain a hydrophobic ring clashing with the amino acids A495 and F496. There are minor differences in the orientation of the others rings, between R and S conformations, due to the chiral carbon located after the furyl group (Figure S1). For hLAP3/KBE009 (R, S) two binding poses, for each diastereomer, with the highest binding energy were picked (−6.7 and −6.6 kcal/mol for S-KBE009 and −6.2 and −5.9 kcal/mol for R-KBE009). As in the case of TcLAP, the binding poses of R- and S-KBE009 with hLAP3 were similar among them, the few differences were related to the chiral carbon (Figure S1). Interestingly, compared to TcLAP, the poses of R and S diastereomers occupied a different space in the active site of hLAP3, they always maintained a hydrophobic ring clashing with I453 and R457, and not with A483 (equivalent to the above-mentioned A495 in the TcLAP sequence).

**Figure 7:**
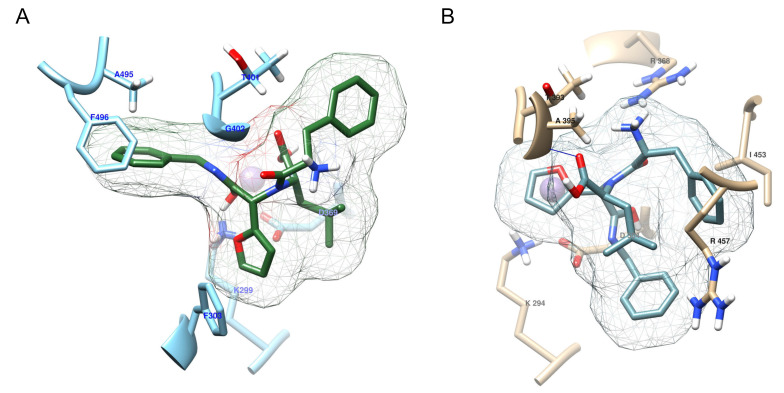
Active site cavity and closed area from TcLAP and hLAP3 showing their interaction with KBE009: (**A**) model of the interaction pattern between TcLAP and KBE009 showing only amino acids (blue) in contact with the peptidomimetic (green); (**B**) cartoon of the hLAP3-KBE009 interaction depicting only amino acid residues (beige) in contact with the compound (turquoise). Only one of the two Mn^+2^ (magenta) is represented. For clarity, only one of the poses of R-KBE009 was represented.

In general, although the KBE009′s structure is voluminous for the catalytic pocket, it could fit relatively well at the entrance of the catalytic domain due to its several rotatable bonds, which allow the compound to adopt different spatial conformations (Figure 6B). The TcLAP-KBE009 complex reported the highest affinity. It showed an autodock vina score for all binding poses between −7.5 and −7.1 kcal/mol and the presence of 1–3 H-bonds (Figure 7A; Table 1), while the hLAP3-KBE009 complex displayed a lower affinity score for all conformations (between −6.7 and −5.9 kcal/mol) and produced 1–2 H-bond (Figure 7B; Table 2). The differences of hydrophilic/hydrophobic ratios at the entrance of the catalytic pockets and their close-environment influence the affinity of the inhibitor-enzyme coupling. KBE009, which is relatively highly hydrophobic, clashed clearly with more hydrophobic amino acids in the active pocket in TcLAP than in hLAP3 (Figure 7A; Table 1). The hLAP3 has a relatively hydrophilic area near the catalytic domain, (Figure 7; Table 1).

### 3.6. KBE009 Meets the Criteria of Drug-Likeness

Drug-likeness predicts whether a compound could be classified as an oral drug candidate. It is based on its structural evaluation and physicochemical properties, according to criteria defined by studying the properties of thousands of compounds mostly present in data banks [31]. This, together with the ADME parameters (absorption, distribution, metabolism, and excretion), are the features to consider at the beginning of the drug discovery process. We used a Swiss ADME server to predict these properties in the KBE009 molecule [31]. Table 2 summarizes the ADME properties and drug-likeness characteristics of KBE009. ADME parameters, such as lipophilicity/water solubility and pharmacokinetics (e.g., gastrointestinal absorption, effect on cytochrome P450 isoenzymes), among others, show favorable results.

At least five different drug-likeness criteria were used to evaluate KBE009. The molecule meets all filters defined by the three following rules: Lipinski’s (Pfizer’s) rule that estimates solubility and permeability [32,33]; the Egan (Pharmacia) rule, which predicts drug absorption in humans [34]; and the Muegge (Bayer) criteria that discriminate drug-like characteristics [35]. KBE009 does not meet only one of the Veber (GSK) rules (based on oral bioavailability measurements in rats for thousands of compounds) and two of the Ghose criteria (based on physicochemical properties and occurrence of functional groups present in drugs) [36,37] (Table 2).

### 3.7. Medicinal Chemistry Analysis of KBE009 Predicts Acceptable Lead-Likeness Parameters

The medicinal chemistry analysis is mostly focused on the compounds per se for the identification of potentially problematic fragments. So emerges the concept of lead-likeness, such as the drug-likeness one described above. With this respect, the structure of KBE009 does not possess predictable toxic or unstable chemical residues, according to the Brenk parameters [38] (Table 2). Besides, no substructures related to a potentially non-specific response, which may lead to promiscuous molecules, were found in the compound (parameter PAINS in Table 2). In general, lead-likeness parameters are acceptable. Only two violations for this filter appeared. The molecular weight, which is not so critical, and rotatable bonds, since it can influence the solubility and the specificity of the compound (Table 2).

## 4. Discussion

Bestatin, also known as ubenimex, is a dipeptide analog that inhibits broad-spectrum metallo-aminopeptidases, including the M1 and M17 families. Bestatin possesses low toxicity in humans and has been used as an adjuvant in the treatment of leukemia and lung cancer [39,40,41]. It has been demonstrated that bestatin affects the TcLAP activity [12,17]. However, *T*. *cruzi* can survive concentrations of up to 300 µM for 6 h [14]. Here, we report the effect of a bestatin derivative, namely KBE009, on the TcLAP activity and the proliferation of the parasites.

KBE009 inhibited the purified recombinant form of TcLAP and the native M17-like activity in crude extracts, at a similar range, confirming that the TcLAP is the main M17 enzyme responsible for the leucyl-aminopeptidase activity in *T. cruzi*, as it was previously reported [12]. This direct inhibitory effect on TcLAP suggests that, at least partially, the deleterious effects of KBE009 observed on the parasite growth are related to its specific action on this enzyme.

KBE009 is also more specific against parasites since it inhibited the *T. cruzi* growth more than that of human cells, with a selectivity index of 4.9. This fact could be explained by the differences in the human and parasite LAPs. To shed light on this possibility, a comparison of TcLAP with a human leucyl aminopeptidase, hLAP3, was performed.

We identified in the human databank, using the TcLAP as a template, more than 10 orthologous sequences, most of them are putative proteins with moderate similarity to TcLAP. This broad repertoire of putative amino acid sequences provides the opportunity for functional redundancy. One of the sequences closely related to TcLAP is the known hLAP3. To gain insights into the specific effect of KBE009 on *T. cruzi*, we run structural analyses of both enzymes. The 3D structures of TcLAP and hLAP3 overlap with high similarity in their secondary structure in the C-termini (where the catalytic center is located).

Interestingly, a docking prediction showed that the TcLAP-KBE009 complex is more stable than its hLAP3 counterpart. KBE009 represented a better geometrical fitting in the active site of TcLAP than that of hLAP3 (especially the R diastereomers, which showed the highest affinity for TcLAP (auto vina score of −7.5 kcal/mol) and the lowest affinity for hLAP3 (−6.2 and −5.9 kcal/mol)). In addition, the inhibition of TcLAP could be supported by the interaction of KBE009 with some residues involved in metal binding (D310, D369, and E371) and in the catalytic activity (K299 and R373). Interestingly, all R- and S-conformations of KBE009 interacted with D369 at the binding site and with K299 at the catalytic pocket.

The concentration range of KBE009 (66–100 µM) required to produce 50% inhibition of the rTcLAP, as well as the native M17-like activity, is comparable to that necessary to reach 50% inhibition of the parasite proliferation (~30 µM). These findings suggest that the anti-Trypanosomal activity of the compound can be, at least in part, associated with the inhibition of the TcLAP. Then, it raises the hypothesis that the enzyme may be essential for the parasite. To date, the essentiality of LAPs in *T. cruzi* has not been reported, and the information on closely related parasitic protozoa is not conclusive. For instance, LAP is essential for *Plasmodium falciparum* but disposable for *T*. *brucei* [12,18,42]. While *T*. *brucei* and *T. cruzi* are more closely related, the former is an extracellular parasite. In intracellular parasites as *T*. *cruzi* and *Plasmodium*, aminopeptidase enzymes could play important roles due to the protein-rich environment where they live. Indeed, M17 and M1 enzymes have been proposed to play a role in the later stages of hemoglobin digestion, an essential process for *Plasmodium* survival [42,43]. For several *Leishmania* species, a kinetoplastid protozoa closely related to *T. cruzi* (which also have an intracellular phase), a single copy of the lap gene has been described [44]. Although the essentiality of this gene has not been addressed yet, it seems to be relevant for the viability of the parasite. The encoded enzyme (M17-LAP), with a high and restricted substrate specificity, represents the most LAP activity in parasite extracts [44]. Inhibition of this enzyme could be deleterious as branched-chain amino acids are essential for *Leishmania* survival [45]. Particularly, the amino acid leucine is a precursor for fatty acids and sterol biosynthesis [46].

Although the concentration that inhibits the enzyme and the one that inhibits parasite proliferation are close, they are not the same. This fact may be due to either an accumulation of the molecule inside parasites or additional effects of KBE009 on the function of other proteins. The increase of KBE009 within the parasites could be favored by its physicochemical properties, such as its lipophilicity/water solubility, and some of the predicted ADME characteristics. Additionally, a rapid intake of the molecule into the parasite could also result from specific transporters at the plasma membrane. In this sense, remarkable examples involving pathogenic protozoa are found in the literature. Aquaglyceroporins (glycerol and water channels) of *T. brucei* and *Leishmania* spp. have been identified as the principal entry pathway for marketed medicaments. Downregulation of these channels causes drug resistance to commercial drugs (pentavalent antimonials, pentamidine, and melarsoprol) [47].

The possibility for the peptidomimetic to interact with other proteins from *T. cruzi* also exists. KBE009 inhibits PfA-M1 and its efficacy against *Plasmodium falciparum* is comparable to that found in this study against *T*. *cruzi* [15]. Therefore, it is reasonable to speculate that the peptidomimetic could act on a PfA-M1 orthologous in *T*. *cruzi*. M1-alanyl-aminopeptidases from *T*. *cruzi* have not been characterized yet. Interestingly, a BLAST search on data banks of different strains of *T*. *cruzi*, using PfA-M1 as a template, retrieved few sequences with e-values around 1 × 10^−14^ that would be putative M1 peptidases. Moreover, PfA-M1 is an enzyme with more substrate promiscuity than the M17 LAP from *P. falciparum* (PfA-M17), due to a much larger cavity of its active site [48] which, in turn, would facilitate the ingress of KBE009 [14]. A similar phenomenon could be expected to occur with an eventual *T. cruzi* PfA-M1 ortholog. In fact, we performed a preliminary study to detect the M1-type activity in protein extracts of *T. cruzi* CL Brener, using 500 µM of the substrate Lys-pNA (typical of the M1- but not the M17-family) [49]. We detected this activity, which was inhibited by bestatin (65%) and KBE009 (71%) at 20 µM of the inhibitors (ongoing experiments). According to our hypothesis, KBE009 could inhibit the *T*. *cruzi* PfA-M1 ortholog more potently than the TcLAP.

For a compound to go through a hit-to-lead process, it is necessary to meet some criteria related to physicochemical properties, solubility, pharmacokinetics, drug-likeness, medicinal chemistry, purity, and suitability for synthesis. In silico analyses showed that KBE009 satisfactorily matches most of these requirements. However, KBE009 does not meet additional criteria for in vitro effectiveness, namely a selectivity index of 10 and an EC50 below 30 µM or 10 µM (varies according to the literature) [16,50]. Although the values obtained are not that far from meeting the criteria, the use of the *T. cruzi* insect forms, epimastigotes, as a first approach to evaluate the efficacy of the compound, establishes some restrictions in the interpretation of the results. A more physiological picture would be represented by the replicative forms of the parasites in mammalian cells, amastigotes.

KBE009 requires some changes to enter a “hit-to-lead” process. The optimization of the peptidomimetic structure, including the decrease of the molecule size and the number of rotatable bonds, is necessary to improve the molecule. A reasonable strategy in a long run would be to study the M1 putative orthologs from *T. cruzi* to define whether this enzyme is an important target of the peptidomimetic, as we hypothesized. It could allow us to orchestrate a better approach for the design of a more effective KBE009 derivative, which can inhibit both peptidases (M1 and M17) from *T. cruzi*. This strategy, including two targets, may decrease the probability that *T. cruzi* rapidly develops resistance against the molecule, as it has already been proposed for malaria [48].

Taken together, the bestatin-based peptidomimetic KBE009 has been investigated as a novel anti-Trypanosomal agent. This compound was moderately active against parasites in a low micromolar range. It was also demonstrated that KBE009 is an inhibitor of the *T. cruzi* M17-LAP consistent with its inhibitory activity on the parasite growth. However, inhibition of other peptidases, mainly the putative *T. cruzi* M1-aminopeptidase, must be studied. KBE009 successfully overcame most of the in silico filters of drug-likeness and lead-likeness required to continue advancing in the development of a drug candidate. However, further experimental optimization of the compound is required to improve its potency against *T. cruzi*. discuss the results and how they can be interpreted from the perspective of previous studies and of the working hypotheses. The findings and their implications should be discussed in the broadest context possible. Future research directions may also be highlighted.

## Figures and Tables

**Table 1:** Amino acids in TcLAP and hLAP3 predicted to interact with KBE009.

N°	TcLAP			hLAP3		
	Residues	Polarity	Type of Interaction	Residues	Polarity	Type of Interaction
1	Lys 299	polar	wdw	Lys 294	polar	wdw
2	Phe 303	Hydrophobic	wdw	Asp 364	polar	wdw
3	Asp 369	polar	wdw	Arg 368	polar	wdw
4	Thr 401	polar	wdw	Thr 393	polar	wdw
5	Gly 402	Hydrophobic	wdw	Ala 395	Hydrophobic	H-bond
5a	Gly 402	Hydrophobic	H-bond			
6	Ala 495	Hydrophobic	wdw	Ile 453	Hydrophobic	wdw
7	Phe 496	Hydrophobic	wdw	Arg 457	polar	wdw

This table corresponds to the interaction of amino acids depict in Figure 6. However, only amino acids that interact with both KBE009 diastereomers (R and S) are represented. wdw, van der Waals.

**Table 2:** Drug-likeness and lead-likeness parameter of KBE009.

	Characteristics	KBE009	Comments
Physicochemical properties	Formula	C_28_H_33_N_3_O_5_	
	Molecular weight (MW)	491.58	
	N° Rotatable bonds	14	
	N° H-bond acceptors	6	
	N° H-bond donors	3	
	Molar Refractivity (MR)	136.10	
	TPSA	125.87	
Lipophilicity	Consensus Log P (average of five different prediction tools)	2.57	meet all filters of drug-likeness
Water solubility	Log S (ESOL/Ali/SILICOS-IT)	−3.25/−3.73/−6.96	soluble/soluble/poorly soluble
Pharmacokinetics	GI absorption	high	
	BBB permeant	no	
	Pgp substrate	yes	
	inhibitor of CYP1A2/CYP2C19/CYP2C9	no	
	inhibitor of CYP2D6/CYP3A4	yes	
Drug-likeness	Lipinski ^1^	yes	
	Egan ^2^	yes	
	Muegge ^3^	yes	
	Veber ^4^	no	1 violation: N°. of rotatable bonds > 10
	Ghose ^5^	no	2 violations: MW > 480, MR > 130
Medicinal Chemistry	PAINS N° alerts	0	
	Brenk N° alerts	0	
	Lead-likeness N° violations	2	MW > 350, Rotors > 7
	Synthetic accessibility	4.66	

^1^ Lipinski (Pfizer) rule estimates solubility and permeability (four drug-like physicochemical and structural features are evaluated: donors and acceptor of hydrogen bond, molecular mass, and octanol-water partition). ^2^ Egan (Pharmacia) rule, a prediction tool of drug absorption in humans, based on literature data of known compounds. ^3^ Muegge (Bayer) criteria, based on functional motifs that help to discriminate drug-like characteristic. ^4^ Veber (GSK) rules are based on oral bioavailability measurements in rats for thousands of compounds. ^5^ Ghose criteria determined by physicochemical properties and occurrence of functional groups present in drugs.

## Data Availability

Datasets generated and/or analyzed in this study are available from the corresponding author on reasonable request.

## Supplementary Materials

The following are available online at www.mdpi.com/article/10.3390/life11101037/s1. Figure S1: Illustration of the superposition from the different poses of R and S diastereomers at the enzyme´s binding site according to the docking using autovina.
