# Supplementary material for: KBE009: A Bestatin-Like Inhibitor of the Trypanosoma cruzi Acidic M17 Aminopeptidase with In Vitro Anti-Trypanosomal Activity
Source: Life (Basel). 2021 Oct 1;11(10):1037. doi: 10.3390/life11101037 (PMC8540442; doi:10.3390/life11101037)
Supplement: Supplementary file 1 [file life-11-01037-s001.zip › life-1370037-supplementary.pdf]

life

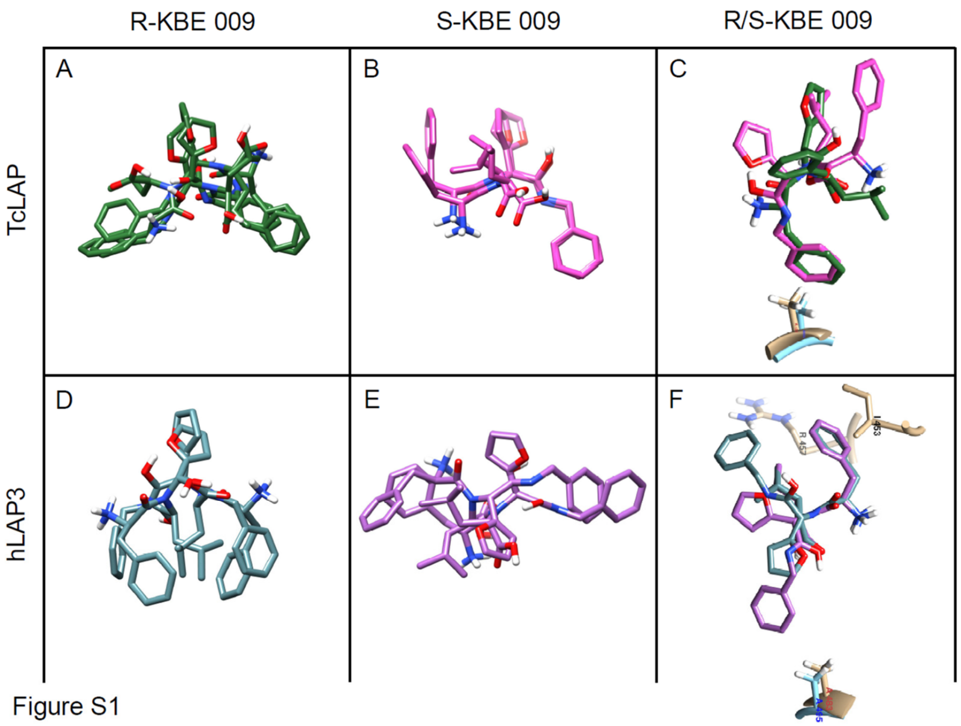

Figure S1. Illustration of the superposition from the different poses of R and S diastereomers at the enzyme´s binding site according to the docking using autovina. The enzyme structure has been removed from the models to facilitate the comparison of R/S KBE009 poses: (**a**) and (**b**) represent poses of the R and S diastereomers at the TcLAP biding site, respectively; (**c**) Representation of one pose of each diastereomer (R- and S-KBE009) overlapped in the active site of TcLAP. For spatial orientation, the amino acid A495 from TcLAP and its counterpart in hLAP3 (A483) are shown; (**d**) and (**e**) Poses of the R and S diastereomers at the hLAP3 biding site, respectively; (**f**) the superposition of one pose of each diastereomer (R- and S-KBE009) in the active site of hLAP3 is represented. For spatial orientation, the amino acid A483 from hLAP3 and its counterpart in TcLAP (A495) are shown. The amino acids R457 and I453 from hLAP3 are also represented as they are relevant for the docking in this enzyme and highlight the shift of the KBE009 in the active site of hLAP3, compared to TcLAP. To demonstrate the difference in the orientation of KBE009 in the active sites of both enzymes [(**c**) and (**f**)] TcLAP and hLAP3 structures were superimposed and some relevant amino acid residues from both enzymes are displayed. Only one of the poses from each diastereomer is represented.
